# Editorial: Parkinson's disease: cell vulnerability and disease progression

**DOI:** 10.3389/fnana.2015.00125

**Published:** 2015-09-15

**Authors:** Javier Blesa, Jose L. Lanciego, Jose A. Obeso

**Affiliations:** ^1^HM Hospitales, Centre for Integrative Neuroscience A.C., Hospital Universitario HM Puerta del Sur, Mostoles and CEU San Pablo UniversityMadrid, Spain; ^2^Center for Networked Biomedical Research on Neurodegenerative Diseases, Institute Carlos IIIMadrid, Spain; ^3^Center for Applied Medical Research, University of Navarra Medical CollegePamplona, Spain

**Keywords:** Parkinson disease, synuclein, dopamine, vulnerability, substantia nigra, striatum

The hallmark of Parkinson Disease (PD) is the degeneration of dopaminergic neurons in the substantia nigra pars compacta (SNc) and the consequent striatal dopamine (DA) deficiency, although it is well recognized that neurodegeneration in PD goes beyond the SNc. Major advances have occurred in recent years on the molecular and pathophysiological basis of PD, however there remain many questions and unknowns regarding SNc cells vulnerability, and the exact significance of Lewy bodies and alpha-synuclein (α-syn) aggregation process regarding disease onset and progression. This Research Topic discuss the etiopathogenesis of PD, presenting a series of papers that provide up-to-date, state-of-the-art information on molecular and cellular mechanisms involved in the neurodegeneration process, neuroimmune pathways, the role of functional and anatomical organization of the basal ganglia as a factor of neuronal vulnerability, the possibility that PD is a prion disease and the cellular response to α-syn aggregation. Understanding the mechanisms underlying vulnerability of dopaminergic midbrain neurons and how pathology becomes widespread are primary objectives of basic and clinical research in PD.

Are dopaminergic and other neurons dying by the same pathogenic mechanisms? Do they all die to the same extent or at the same rate? What are the molecular determinants of susceptibility to the disease? To gain insights into these questions, researchers mainly rely in animal models. Blesa and Przedborski (2014) provide a summary of the current knowledge of *in vivo* models of PD. Whereas PD can be sporadic, genetic or possibly related with toxic/infectious agents, a differential pattern of cell loss among midbrain dopaminergic neurons is observed regardless of disease etiology suggesting that differential dopaminergic neuron vulnerability does not depend on the factor triggering PD “*per se*” but on intrinsic properties of these specific cell groups. Here, Brichta and Greengard (2014) provides an update review on the molecular basis underlying differential vulnerability of midbrain dopaminergic neurons in PD. For example, for many years many studies have suggested calbindin (CB) as a marker to distinguish between midbrain dopaminergic neurons with different susceptibility to degeneration in PD. Although CB dopaminergic neurons seem to be less prone to MPTP-induced degeneration, Dopeso-Reyes et al. (2014) clearly demonstrated that these neurons are not giving rise to nigro-striatal projections and indeed CB-ir/TH-ir neurons only originate nigro-extrastriatal projections. This data sustain the presence of a potential imbalance between the nigro-striatal and nigroextrastriatal systems in advanced diseases states. Also, Afonso-Oramas et al. (2014) revealed that midbrain dopaminergic axons are in close apposition to striatal vessels and perivascular astrocytes in rats and monkeys. The relative weight of this “vascular component” within the meso-striatal pathway suggests a role in the pathophysiology of PD.

Aging is another major risk factor for developing PD. Rodriguez et al. (2014) reviewed similarities between neurodegeneration in PD and aging. The progressive course of aging and PD could be induced by the same multi-factorial etiology, including astrocytic and microglia alterations, anomalous action of different proteins, mitochondrial disturbances, alterations of the mitophagy or the ubiquitin-proteasome system and oxidative stress. Proteins involved in PD such as α-syn, PINK1 or DJ-1, are also involved in aging. All these mechanisms of degeneration are review here giving an update of the classical pathways, the biochemical and molecular events that mediate DA neuronal vulnerability, and the role of PD-associated gene products in modulating cellular responses to oxidative stress (Blesa et al., 2015). Additionally, Labandeira-García et al. (2014) discuss the role of renin-angiotensin system in oxidative stress, aging and inflammation in the nigrostriatal dopaminergic system. Inflammation is indeed a major characteristic feature of the SNc in PD mainly as a consequence of neuronal death. Herrero et al. (2015) review the role of inflammation and glucocorticoids in PD while Cebrián et al. (2014) review the neuronal MHC-I expression in the SNc and its implications in synaptic function, axonal regeneration in PD and other brain diseases.

The dopaminergic neurons of the SNc project primarily to the striatum, but also provide significant innervation of other basal ganglia nuclei and the thalamus. Villalba et al. (2015) discuss evidence for synaptic glutamatergic dysfunction and pathology of cortical and thalamic inputs to the striatum and subthalamic nucleus in models of PD. The altered neuronal firing activity of the basal ganglia and other nuclei contribute largely to parkinsonisms. Galvan et al. (2015) reviewed the current knowledge of the electrophysiologic changes at the single cell level, the level of local populations of neural elements, and the entire basal ganglia-thalamocortical network in PD, and discuss the possible use of this information to optimize treatment approaches. Neuroprotection by endogenous glial cell-derived neurotrophic factor (GDNF) stimulation has been suggested as one of the potential preventive therapies in PD for many years. In this issue d'Anglemont de Tassigny et al. (2015) summarize current knowledge on brain GDNF delivery, homeostasis, and its effects on SNc neurons and discuss the therapeutic potential of endogenous GDNF stimulation in PD.

Formation and accumulation of misfolded α-syn aggregates are a central and very hot topic of PD research currently. Several authors review and discuss here the importance of this protein, it's role in different cellular domains (Guardia-Laguarta et al., 2015), the pathophysiological mechanisms connecting α-syn and lysosomal dysfunction in neuronal cell death (Bourdenx et al., 2014), how this protein can undergo a toxic conformational change, spread from cell to cell and from region to region, and initiate the formation of aggregates (Recasens and Dehay, 2014), and its possible relation to other neurodegenerative diseases like progressive supranuclear palsy (Erro Aguirre et al., 2015).

While all the features summarized above play a significant role in nigro-striatal neurodegeneration, it is unlikely that the origin of neurodegeneration in PD could be tight to a single pathogenic mechanism, hence the importance of defining markers and features of neuronal vulnerability. Obeso's group introduces here the interesting hypothesis that Parkinson's disease could be related and ultimately be the consequence of human multi-tasking behavior (Hernandez et al., 2015). Thus, the caudal region of the striatum has been associated with habitual behavior, consequently the differential loss of DA from this region provides the pathophysiological substrate for the early impairment of automatic movements (walking, writing…) and probably increased functional demand during multiple and simultaneous tasks performance.

In sum, understanding the mechanisms responsible for intrinsic SNc neuronal vulnerability is mandatory to progress in stopping neurodegeneration in PD. We trust that this Research Topic will spark new ideas and foster further advances in PD.

## Conflict of interest statement

The authors declare that the research was conducted in the absence of any commercial or financial relationships that could be construed as a potential conflict of interest.
